# Mucosal Melanoma: Epidemiology, Clinical Features, and Treatment

**DOI:** 10.1007/s11912-023-01453-x

**Published:** 2023-09-29

**Authors:** Maria Chiara Sergi, Elisabetta Filoni, Giacomo Triggiano, Gerardo Cazzato, Valeria Internò, Camillo Porta, Marco Tucci

**Affiliations:** 1 Department of Interdisciplinary Medicine, Oncology Unit, University of Bari “Aldo Moro”, P.za Giulio Cesare, 11, 70124 Bari, Italy; 2 Section of Molecular Pathology, Department of Precision and Regenerative Medicine and Ionian Area (DiMePRe-J), University of Bari “Aldo Moro”, 70124 Bari, Italy; 3 Oncology Unit San Paolo Hospital, Bari, Italy

**Keywords:** Mucosal melanoma, Immunotherapy, Targeted therapy, cKIT mutation, BRAF mutation

## Abstract

**Purpose of Review:**

Summarize the writings published in the last years on the management and novel therapies of mucosal melanoma (MM).

**Recent Findings:**

New research has demonstrated a difference between MM and cutaneous melanoma (CM) in their genomic and molecular landscapes, explaining the response's heterogeneity. Immunotherapy and targeted therapy have limited benefit, but novel therapies are rapidly expanding.

**Summary:**

MM is aggressive cancer occurring in gastrointestinal, respiratory, or urogenital mucosa; whose incidence is greater in the Asian population. The etiology and pathogenesis remain unclear since UV exposure is not a proven risk factor as in cutaneous melanoma. In contrast to CM, lesions on the mucosal surface are less likely to be recognized early; therefore, the disease is diagnosed in an advanced stage. Clinical manifestations, such as bleeding or pain, can help to detect this tumor, although the prognosis remains unfavorable with an overall 5-year survival rate of less than 20%. The mutational landscape of MM includes mutations of BRAF and NRAS, as well as mutations in the c-KIT/CD117 gene (in 50% of patients), thus limiting therapeutic interventions to immunotherapy. However, clinical studies show less responsiveness to immunotherapy compared to CM, therefore novel therapeutic strategies targeting new molecules are needed to improve the survival of patients with MM.

## Introduction

Melanoma develops from melanocytes that originate from the neural crest and migrate to many tissues during embryogenesis [1]. The biology and clinical outcome of cutaneous melanoma (CM) is different from that of mucosal or ocular melanoma [2]. MM accounts for 1,3% of all melanomas, and develops in non-sun-exposed areas, including the mucous membranes of the respiratory, gastrointestinal, and genitourinary tracts.

To date, the risk factors are poorly understood and the pathogenesis remains unclear [3], although an association with human papillomavirus and herpesvirus has been described [1]. In contrast to CM, the diagnosis of mucosal evolutive lesions is more difficult and MM is therefore likely to be detected at a late stage [2]. As a result, MM is usually diagnosed late, and malignant cells are often surrounded by a rich vascular and lymphatic network, which is mostly considered to be the cause of aggressive behaviour and poor prognosis. [2]. The molecular landscape includes a lower incidence of BRAF oncogene mutations but a high frequency of KIT mutations, suggesting a distinct genetic origin with respect to CM [2]. The overall five-year survival rate for MM is 10–20% [1]. However, new therapeutic strategies have the potential to improve the outcome of MM, which has a poor response to immunotherapy [1]. Here, we present a comprehensive review of different primary MM, their epidemiological and clinical features, and therapeutic strategies.

## Epidemiology

Mucosal melanoma is a rare and aggressive subtype, accounting for 0.8–3.7% of all melanomas in Caucasians [1]. In contrast to CM, whose incidence is steadily increasing, the incidence of MM is almost stable and is more likely to affect women than men (2.8 versus 1.5 per million) [1]. The incidence of MM is also influenced by age, with more than 65% of patients over 60 years of age and less than 3% under 30 years of age. Racial differences in incidence have been reported. Although ethnicity influences the onset of MM, the absolute incidence remains higher in Caucasian populations [1]. The incidence is higher in Caucasians, but Asians have a higher incidence of MM arising from the anorectal tract, whereas non-Hispanic whites have a higher incidence of genitourinary MM [2, 4]. Finally, Hispanics most commonly present with MM of the head and neck [3].

## Etiopathogenesis and Clinical Features

Melanocytes are not found exclusively in the epidermis, iris, and hair but also in the inner ear, nervous system, heart as well as mucous membranes [1, 2]. Based on recent studies, melanocytes produce many molecules in consequence of UV exposure as cytokines, melanocortin, amines, and nitric oxide (NO), thus promoting melanogenesis [1]. As recently reported, keratinocytes, lymphocytes, fibroblasts, mast cells, and endothelial cells may represent potential targets of these secretory compounds. In addition, α-MSH controls the production of NO by melanocytes while and melanocortins promote other signals that are considered key regulators of skin and mucous membrane homeostasis [2, 3]. Notably, MM is not associated with UV exposition and those arising from the anorectal tract tend to affect patients with red hair and poor tanning history [5]. Because the pathogenesis of MM is unrelated to typical carcinogens, there is poor evidence to support a link between cigarettes, formaldehyde, or exposure to cancer-causing viruses (i.e., papillomavirus, herpes virus, or polyomavirus) [2, 6]. For example, regarding the head and neck MM, melanocytes could play a key role in the metabolization of polycyclic aromatic hydrocarbons [7, 8]. However, the origin and pathogenetic mechanisms remain still undefined [3]. In the context of clinical presentation, the MM are lesions with irregularity of borders, altered pigmentation, and elevated areas. In contrast to many CM, which in general can be easily detected, lesions occurring in the mucosal surfaces are less likely to be detected early in their evolution [2]. On the other hand, the clinical symptoms depend on the site of onset of melanoma, and in the case of head and neck lesions, blood streaks can be observed in the nasal secretions, epistaxis, proptosis, diplopia, and pain [2].

### Mucosal Melanomas of the Respiratory Tract

Mucosal melanomas of the respiratory tract are rare and arise from melanocytes of the ectodermal mucosa. The nasal cavity and sinuses are common sites (about 4% of all sino-nasal neoplasms) [2] whereas it is extremely rare in the larynx and tracheobronchial tract [9]. By contrast, the lung is frequently colonized by melanoma metastases of cutaneous, ocular, or mucosal origin [9].

Most patients suffer from nasal obstruction, visible mass and epistaxis that in some cases cause pain, morphological changes of the face, ptosis, and diplopia. Laryngeal mucosal melanoma is extremely rare and there are approximately 60 cases reported [9]. This subtype affects generally males in the sixth and seventh decade [1]. It causes hoarseness, followed by throat irritation but also sore throat, dysphagia, neck swelling, and pain [10] Primary tracheal melanoma is a very rare cancer with four cases reported showing clinical manifestations that resemble other tracheal tumors as airway obstruction, dyspnea and stridor as well as hoarseness, cough and hemoptysis. In most cases, the treatment consists of palliative surgery for restoring the airway or tracheal resection [11]. Primary lung melanoma is an extremely rare cancer with around 30 cases described whereas the lung is a common metastatic site. [12]. Endobronchial growth is often peripheral and is detected by bronchoscopy as a pigmented or non-pigmented lesion [1].

### Mucosal Melanomas of the Gastrointestinal Tract

Mucosal melanoma frequently affectes the oral cavity and the anal canal (33% and 31% of patients respectively), while other sites such as the esophagus, stomach, small intestine, or gallbladder are rarely involved. Only 14% of patients with primary MM of the gastrointestinal tract are under the age of 50, and about 50% are over the age of 70. [12]

The incidence of oral MM is reported to be 0.2 per million [13], and it develops as a de novo asymptomatic melanocytic lesion with the onset of bleeding or pain. Melanoma of the oesophageal mucosa is rare, accounting for only 0.1–0.2% of all oesophageal neoplasms [14]. It is frequently located in the central and distal esophagus, whereas only in 10% of cases is proximal, in 60 years old male patients: males actually have approximately twice the incidence as females. Symptoms include dysphagia but also retrosternal pain, weight loss, and rarely hematemesis or melaena.

On endoscopy, lesions appear hyperpigmented and ulcerated with satellite nodules [15]. The stomach is rarely involved in melanoma and only a few cases have been reported. Symptoms are non-specific and include abdominal pain, weight loss, upper gastrointestinal bleeding and anaemia [3]. Although primary small bowel melanoma is very rare, it is the most common site of gastrointestinal melanoma metastases. Patients typically present with nausea, vomiting, anorexia, abdominal pain, weight loss, gastrointestinal bleeding with secondary anaemia, and intussusception. [1].

The most common type of GI melanoma, with an estimated incidence rate of 0.4 per million, is anorectal mucosal melanoma [2]. It is the third most common site after cutaneous and ocular melanoma due to malignant transformation of anal and rectal melanocytes. Anal and rectal melanoma occurs most commonly in the seventh decade of life, with a higher incidence in women and a prevalence in Caucasians [1, 16]. It causes rectal bleeding, pain or discomfort and is often diagnosed at an advanced or metastatic stage. The tumor is usually polypoid, ulcerated with or without pigmentation. Primary colorectal melanoma is rare. The average age of patients is 60 years, regardless of sex, with a predilection for the right colon [17]. Abdominal pain and weight loss are common complaints. Primary biliary tract melanoma is extremely rare and may arise in the gallbladder or bile duct. Presenting symptoms are gallstones or cholecystitis, which cause obstructive jaundice, pain, itching and dark urine. Biliary tract melanomas are often metastatic and present as flat, pigmented lesions, whereas primary lesions are often single polypoid lesions on macroscopic examination [18].

### Mucosal Melanomas of the Urogenital Tract

Urogenital tract melanoma is more common in females of Caucasian ethnicity in about 90% of cases [19]. They develop in the vulva, vagina, cervix, urethra, and urinary bladder. The female genital tract accounts for 18% of MM and 3% of those of the urinary tract; the most common site is vulvar (76.7%) followed by vaginal (19.8%), while cervical melanoma is the least common [1]. The second most frequent vulvar cancer is vulvar melanoma. [19] Symptoms include bleeding, lumps or masses in the vulvar area, itching, pain or irritation, discomfort, and discharge. [20] Vaginal melanoma affects 80% of postmenopausal older women with an average age of about 60 years are affected by vaginal melanoma [20]. Pain, mass lesions, and vaginal bleeding and discharge are the most typical presenting symptoms [21]. It appears macroscopically as a variety of pigmented lesion that is fragile, easily bleeds, and ulcerated in half of the cases [1]. Melanoma of the cervix is extremely rare [22] and appears as a variously pigmented or amelanotic exophytic cervical mass. Melanoma of the urethra accounts for about 4% of all urethral malignancies [23] and mostly affects elderly women in the distal tract of the urethra.

In about 20% of cases, MM of the urethra is amelanotic and has polypoid growth, therefore is mistaken for a urethral polyp, mucous prolapse, or urothelial tumor [24]. In the literature, 20 cases of urinary bladder melanoma are reported, underlying the rarity of this neoplasm. Clinical manifestations include hematuria and dysuria and it is often locally advanced at diagnosis [25, 26].

## The Genetic Landscape of Mucosal Melanoma

Non-cutaneous melanoma shows different genetic alterations compared to CM, without a clear molecular target pathway [1, 8].

Specifically, MM has a lower burden of somatic mutations as well as UV-related genetic mutations that are widely described in CM [27].

As in cutaneous and acral melanoma, the mutational profile categorizes MM into different molecular groups: BRAF-mutated; RAS-mutated; NF1-loss; and triple wild-type (absence of mutations in BRAF, RAS and NF1; but the presence of KIT mutations and/or amplifications) [28].

**Fig. 1:**
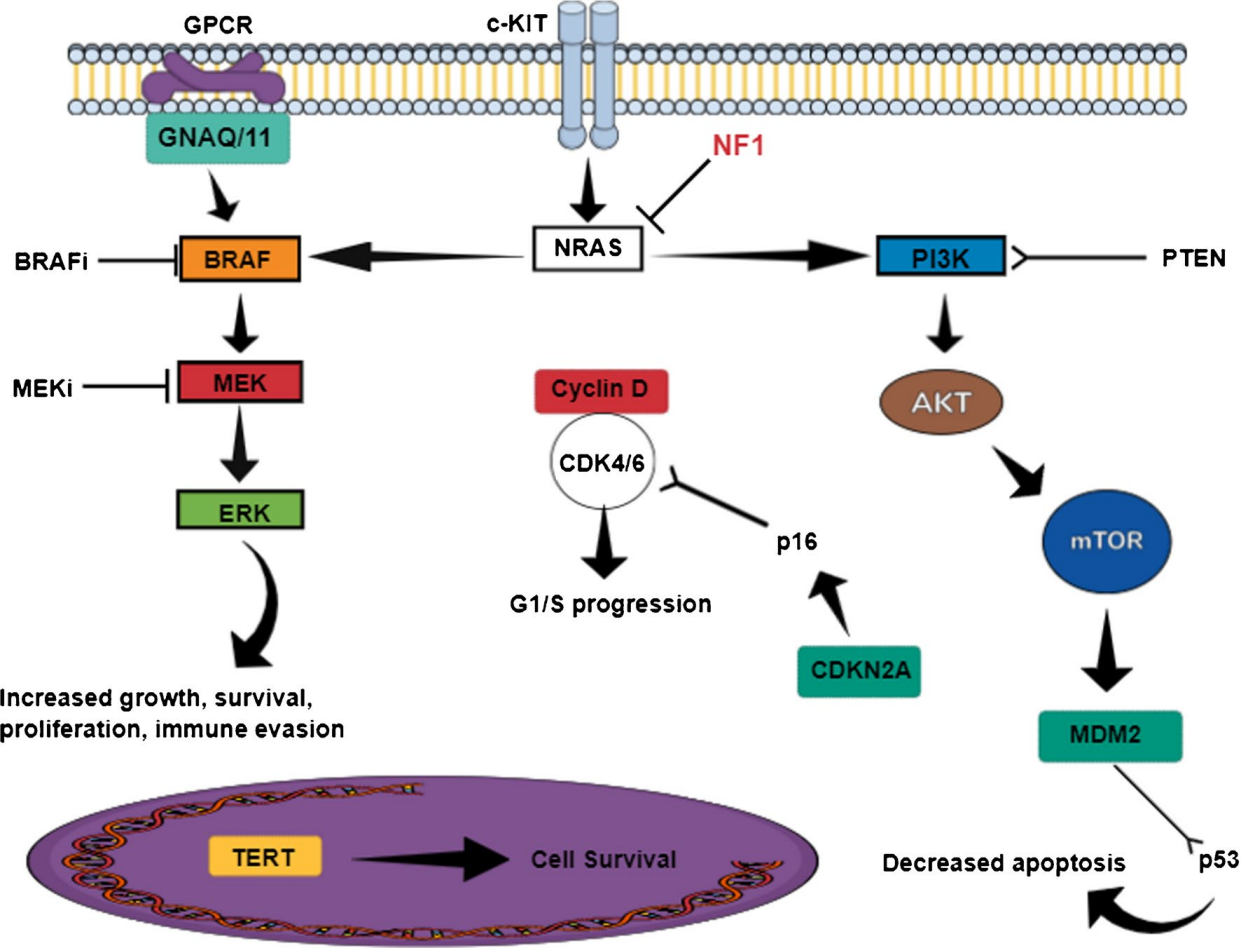
Main signaling pathways involved in melanoma proliferation: activation of the MAPK signaling cascade and PI3K-AKT-mTOR pathway dysregulation by several oncogenes. Also, TERT promoter mutation is linked to the survival, invasion, and metastasis of melanoma

The main pathways involved in the development of MM are summarised in Fig. 1.

The main subtype is characterized by an activating mutation of BRAF, which is involved in MAPK (mitogen-activated protein kinase) signaling, followed by the NRAS mutated group [28].

Activation of MAPK is a major cause of cell proliferation in melanomas that arise on non-chronically sun-damaged skin [29]. However, BRAF/NRAS-activating mutations are less frequently mutated in MM compared to CM [30]: 6% and 8%, respectively, versus 50% and 28% in CM, with a variable incidence depending on the anatomical district of melanoma occurrence [31].

According to Beaudoux’s meta-analysis, head and neck MMs have the highest percentage of BRAF mutations, while NRAS mutations are more frequently mutated in MMs of the urinary tract. Surprisingly, the BRAF and NRAS mutations in MM are more similar to those found in tumors such as lung cancer, suggesting an unidentified link to some genotoxic agents [32].

Indeed, MM may present atypical mutations of BRAF (non-V600) and NRAS (located on codons G12, G13, as well as codon Q61), uncovered in patients affected by a more aggressive disease, not responsive to traditional BRAF and MEK inhibitors, but possibly sensitive to next generation of MEK inhibitors [33].

The third subtype, NF1 loss is around 16% of MM [34] and determines the constitutive activation of Ras proteins. Interestingly, co-mutation of NF1 and KIT is found in 32% of MMs (compared to only 4% for CMs), resulting in upregulation of MAPK cascade [2, 35].

KIT/CD117 gene aberrations predominate in MM as opposed to CM [8, 34]. At least 31 mutations involving *c-KIT* affecting exons 9, 11, 13, 17, and 18 have been described [36] in metastatic melanoma. Some research revealed that the disease’s early and late stages are both characterized by c-KIT immunoreactivity [36], but whether mutations occur at the onset of melanoma or during the advanced stage due to mutation accumulation, remains unclear. Moreover, Gong et al. showed that similar to BRAF and NRAS mutations, the frequency of c-KIT mutation in melanoma is influenced by the anatomical site of the primary tumor: genitalia and extremities having the highest mutation rate (of 26 to 35%), anorectal melanoma expresses in 35% of cases cKIT mutations, while in sinonasal tract cKIT mutations are observed in 22% of MM [37].

As mentioned above, cKIT mutations distinguish the subtype of triple-negative melanoma, often associated with the activation of splicing factor 3b subunit 1 (SF3B1), a protein involved in RNA splicing and transcriptomic regulation (with a higher trend of R625C and R625H mutations in vulvovaginal and anal or rectal melanomas) [36] and Telomerase Reverse Transcriptase (TERT) promoter mutations. The overexpression of TERT, which is present in about 30% of head and neck MMs, gives cells the ability to become immortal and therefore aggressive behavior [38, 39].

St.rikingly, some observations uncovered that Sprout-related EVH1 domain-containing protein 1 (SPRED1) loss promotes cell proliferation in KIT-driven melanoma, as a result of SPRED1's function as a tumor suppressor in the MAPK pathway. According to previous studies, SPRED1 acts downstream of KIT but upstream of RAF by directly repressing Ras [39]. The MAPK signal induced by KIT activation may be significantly enhanced by SPRED1 loss, implying the possibility that the co-existence of SPRED1 loss and KIT-activating mutations is linked to resistance to KIT inhibitors [40, 41].

Besides, triple wild-type MMs tend to have CCND1/cyclin D1 or cyclin-dependent kinase (CDK) 4 amplification, acting as an alternative driver in BRAF or NRAS-mediated proliferation signaling mechanisms [2] through activation of MAPK and PI3K pathways. This explains a lack of response to antitumor therapy and a worse prognosis for this melanoma subtype [42].

As reported by Wang et alresearch’s, older MM patients who have higher levels of CDK4 protein expression have lower 3-year survival [43]. Other uncommon mutations are in TP53 (9% of cases), ATP-dependent helicase (ATRX) (in 6% of MM), and Phosphatase and tensin homolog (PTEN) inactivation through methylation of the PTEN promoter, a rare occurrence involved in the development of Sinonasal MM [44].

PTEN loss and amplification of CDK4/6 pathways upset the immune system's delicate balance, resulting in immunosuppression and decreased response to immunotherapy [43].

Moreover, the low mutational load described in MM as well as poor expression of death ligand 1 (PD-L1) compared to CM, limits T cell activation contributing to immunotherapy resistance [39, 45].

In conclusion, several reports identified mutations *GNAQ/11* in 9.5% of patients with MM, without clear therapeutic implications. *GNAQ* and *GNA11* are involved in many physiological and pathological processes such as hydrolysis’s regulation of GTP [46]. Mutations of *GNAQ* and *GNA11* are relatively low (4.6% and 4.9%, respectively) for MM, unlike the data for uveal melanoma characterized by a frequency of GNAQ mutation ranging from 36 and 53%. Furthermore, metastatic MM patients bearing GNAQ/11 mutations showed a shorter median survival than wild-type, supporting a probable correlation with clinical progression. [47].

Hence, MMs have a uniquely complex genomic profile that influences their pathogenesis and aggressiveness, explaining the poor response to systemic therapies.

## Surgical Treatment and Systemic Treatment

### Local Disease

Radical tumor excision is the first approach for locally advanced MM with disease-free surgical margins [48]. However, the anatomical complexity of the region, the metastatic stage at diagnosis, and proximity to vital structures (especially for head-neck tumors) make it difficult to achieve radical surgery [46, 48]. Although challenging surgery procedures, local recurrence is common, particularly in cases of inadequate resection, vascular invasion, and large tumor size [49, 50]. In the head and neck, the median time to first recurrence is 6 to 12 months [51]. Surgery can be paired with adjuvant irradiation to improve local control in patients with clinical lymph node involvement or extracapsular extension [52], although the radiotherapy efficacy is debatable because local failure occurs in half of the patients, and overall survival benefit has not been observed. Sentinel lymph node biopsy (SLNB) is reserved for vulvar and anal melanoma, but its role is controversial because inguinal lymphadenectomy does not improve survival. Therefore, bilateral nodal dissection is performed in patients who have a clinically node-positive disease, eligible for further adjuvant systemic treatment [53].

Due to the diagnostic delay and advanced stage of presentation, systemic therapy is often palliative while adjuvant treatment shows a marginal role. To date, BRAF mutation is mandatory in stage III or IV melanoma [54].

In BRAF wild-type tumors, adjuvant immunotherapy with antibodies targeting PD-1, nivolumab, or pembrolizumab, is a viable choice. In BRAF-mutated MM, targeted therapy with BRAF and MEK inhibitors is an option, in addition to adjuvant immunotherapy [55].

Nevertheless, most trials have enrolled patients with cutaneous melanoma, except for CheckMate 238, whereby 29/906 patients with MM were included, to evaluate adjuvant Nivolumab, or Ipilimumab in resected stage IIIB, IIIC, and IV melanoma. Interestingly, Japanese subgroup analysis revealed a low efficacy of Nivolumab in mucosal melanoma than in cutaneous melanoma (51.7% in the nivolumab arm vs 41.7% in the ipilimumab arm) [56]. Despite the effectiveness advantage of the combination of nivolumab and ipilimumab in CheckMate 067 [57] for metastatic melanoma, no such recurrence-free survival improvement was observed in the adjuvant setting in CheckMate 915, with lower ipilimumab dose in both cutaneous and mucosal melanoma (7% of the population in the combination arm) [58].

Adjuvant chemotherapy was investigated in a phase III trial of 204 resected MM patients. This trial evaluated temozolomide plus cisplatin versus one year of interferon-alfa-2b [59]. In preliminary evidence, at a median follow-up of 24 months, chemotherapy improved relapse-free survival (median 15.5 versus 9.5 months) and distant metastasis-free survival (median 16.8 months versus 9.6 months). To date, chemotherapy should not be considered the standard of care but exclusively offered to patients not suited for immunotherapy or targeted therapy [60]. Regardless of local diseases, neoadjuvant therapy is evolving. At a median follow-up of 59 weeks, neoadjuvant toripalimab (a humanized antibody targeting PD1) plus axitinib (a selective inhibitor of vascular endothelial growth factor [VEGF] receptor tyrosine kinases 1, 2, and 3) revealed a complete pathologic response rate of 29 percent in a phase II study of 21 patients with resectable MM, representing a new opportunity [61].

### Unresectable and Metastatic Disease

In patients with metastatic MM lacking a BRAF mutation, a combination of immunotherapy has provided a promising therapeutic approach, albeit clinical studies show less responsiveness compared to cutaneous melanoma [62]. A 5-year subanalysis of the Checkmate 067 trial about mucosal melanoma patients reported a considerably higher objective response rate (ORR) (43% vs. 7%), and Overall Survival (OS) rate (36% vs. 7%) in 79 patients with MM treated with combined Nivolumab and Ipilimumab vs ipilimumab alone [63], while Nivolumab monotherapy was associated with 30% of ORR and 17 % of OS rate. This data is confirmed by D’Angelo et al. pooled analysis of five trials (CA209-003, CA209-038, CheckMate-037, CheckMate-066, and CheckMate-067). Despite the median reduction in tumor burden being -34.2% for combination therapy in the subgroup of MM as compared to cutaneous melanoma patients, ORR and PFS are lower (37 vs 60% and 5.9 versus 11.7 months, respectively). Notably, the response rate is higher in patients with an assessment of PDL1 > 5% (53.3% *versus* 12.2% in PD-L1 < 5).

Regarding safety, the most common immune-related events in grades 3 or 4 were rash and diarrhea, higher for combination therapy [58, 64].

A regimen with pembrolizumab was assessed in several studies, confirming Nivolumab data of ORR (19%), the median PFS (2.8 months), and the median OS (11.3 months) [65].

Interestingly, clinical outcomes in Asian patients are less favorable than in Caucasian individuals.

Keynote 041 phase Ib study, evaluating the safety of pembrolizumab in Japanese patients with metastatic melanoma, showed an ORR of 25% for MM [66]. Moreover, in the Keynote-151 trial and Polaris-01 trial, which investigated pembrolizumab and toripalimab, in the Chinese population with advanced/metastatic MM, the anti-PD1 therapy achieved an ORR of 13.3% and 0%, respectively [67]. Probably, these results reflect the immunotherapy resistance in the Asian group, which had a higher rate of KIT mutations, in addition to lower expression of PD-L1, and lower tumor mutational burden [68–70].

Based on c-KIT mutations, agents targeting c-*KIT* have been examined in mucosal melanoma. According to the results of a retrospective study investigating imatinib's (a c-KIT inhibitor) effectiveness in patients with MM who had KIT mutations or amplification., ORR observed was 21.9%, the overall disease control rate of 60%, and an OS of 13 months and PFS of 4 months [71].

In this regard, the *KIT* exon 11 and 13 mutations demonstrate sensitivity to KIT inhibitors, while KIT exon 17 mutations and *KIT* amplifications show no response to imatinib or similar drugs [72]. Another tyrosine kinase inhibitor Dasatinib, targeting mutation in cKIT exon 11-L572P, has been assessed in the ECOG-ACRINE2607 trial, administered 70 mg twice daily in mucosal, acral, or vulvovaginal melanoma. The results were unsuccessful because PFS and OS were low regardless of cKIT status or subtypes [73]. Thus, Imatinib remains a treatment option for patients with a *KIT* mutation who progressed or suffered adverse events after immunotherapy.

Furthermore, the combination of BRAF and MEK inhibitors is less effective in MM, probably as a consequence of NF1 loss of function mutations or deletions linked to BRAF inhibitor resistance, while no effect of NRAS mutation on metastatic melanoma characteristics or outcomes of checkpoint inhibitor immunotherapy is noted [75].

Data from the Italian Melanoma Intergroup underlie the predictive role of intratumoral CD8 + T cells in targeted therapy in terms of PFS and OS [75].

Another promising therapeutic strategy includes targeting NF1. NF1 mutation is associated with better response to immune checkpoint inhibitors, [76] but also worse overall survival for patients with NF1 WT. Jour et al. found a high expression of MK167 (Marker of Proliferation Ki-67) and CDC20 in these patients, thus suggesting a relation between the nuclear accumulation of CDC20 transcripts and worse prognosis. In this study, MAPK pathway and CDC20 co-inhibition resulted in cytotoxic and cytostatic effects, decreasing CDC20 expression in many NF1-mutant cell lines, supporting the idea that inhibition of CDC20 and MAPK pathways may be a new option for NF1 mutated melanoma [77].

## Conclusions and Future Perspectives

Due to the less effectiveness of immunotherapy compared to cutaneous melanoma, numerous ongoing trials are exploring new combination therapies (Table 1). The combination of anti-angiogenic therapy and immunotherapy results in increases in the number of TILs and inhibits tumor growth in murine cancer models [78]. A single-center, phase Ib trial assays the preliminary efficacy and safety of toripalimab in combination with axitinib in patients with advanced Melanoma [79, 80]. Twenty-nine Asian patients with MM received axitinib and toripalimab every 2 weeks, 14 patients had a partial response, the median OS still not reached after (at 1.5 years of follow-up), and a longer PFS and a better ORR were found in PD-L1-positive tumor.

**Table 1:** A summary of clinical studies (recruiting, active or not yet recruiting) for the perioperative and advanced setting of mucosal melanoma (sources: clinicaltrials.gov)

Therapeutic regimen	Molecular target(s)	Clinical phase	Sample size	Primary outcome	Identifier
Neoadjuvant Setting					
Pembrolizumab Plus Lenvatinib	VEGFR1, VEGFR2, VEGFR3, FGFR1-4, PDGFRα, KIT, RET; PD-1	II	44	Change in immune cell expression of HIF1 and immune cell densities; Pathological response rate	NCT05545969
Pembrolizumab plus IMRT	PD-1	II	50	DFS	NCT03313206
Adjuvant Versus NeoAdjuvant Pembrolizumab	PD-1	II	336	Event-free survival	NCT03698019
Nivolumab alone versus Nivolumab plus Ipilimumab versus Nivolumab plus Relatlimab	PD-1; CTLA-4; LAG3	II	53	Pathologic response	NCT02519322
Toripalimab plus Axitinib	PD-1; VEGFR 1–2-3; PDGFRβ and c-Kit	II	30	Pathological response rate	NCT04180995
Adjuvant Setting					
Ipilimumab Plus Nivolumab	PD-1; CTLA-4	II	36	RFS	NCT03241186
Toripalimab plus Temozolomide	PD-1; DNA	II	294	RFS	NCT04462965
Nivolumab alone vs Nivolumab plus Cabozantinib	PD-1; MET, RET, AXL, VEGFR2, FLT3, and c-KIT	II	99	Efficacy of adjuvant nivolumab alone vs combination therapy	NCT05111574
Hypofractionated radiation therapy plus Pembrolizumab	PD-1	II	16	Local tumor control rate	NCT04318717
Dabrafenib plus Trametinib	BRAF; MEK;	NA	150	RFS	NCT04666272
Advanced Setting					
Toripalimab plus chemotherapy plus Endostar	PD-1; M1 macrophages	II	31	PFS	NCT04472806
Atezolizumab plus Bevacizumab	PD-L-1; VEGF-A	II	43	ORR	NCT04091217
Oral Decitabine/Cedazuridine plus Nivolumab	PD-1; cytidine deaminase; nucleoside metabolic	I/II	30	Safety of DEC-C	NCT05089370
Nemvaleukin Alfa Subcutaneous vs Intravenous	IL-2R	II	110	Centrally-assessed ORR	NCT04830124
Nivolumab plus Axitinib and Ipilimumab or SBRT in select progressors	PD-1; CTLA-4; VEGFR1, VEGFR2, VEGFR3, PDGFRβ and c-Kit	II	20	Best objective response	NCT05384496
APG-115 plus Pembrolizumab	MDM2; PD-1	I/II	224	Maximum Tolerated Dose; Recommended Phase II Dose, Overall Response Rate	NCT03611868
[212Pb] VMT01	MC1R Receptor	I/II	52	Treatment-related adverse events; DLTs, ORR	NCT05655312
Aldesleukin plus Pembrolizumab	T cells (IL-2); PD-1	II	65	Best overall response rate	NCT02748564
Neoantigen Peptide Vaccine Plus Nivolumab and Poly ICLC	T cell; PD-1;	I	20	Incidence of adverse events	NCT05098210
Tebentafusp alone vs Tabentafusp plus durvalumab vs Tabentafusp plus tremelimumab vs Tabentafusp plus tremelimumab plus durvalumab	PD-1; CTLA-4; gp100	I/II	312	DLT and ORR	NCT02535078
Tebentafusp alone vs Tabentafusp plus pembrolizumab	PD-1; gp100	II/III	460	ctDNA reduction on treatment relative to baseline; Overall Survival	NCT05549297
Camrelizumab Plus Anlotinib Plus nab-Paclitaxel	Microtubules; PD-1; VEGFR	II	66	ORR	NCT04979585
Recombinant Vesicular Stomatitis Virus-expressing Interferon-beta and Tyrosinase Related Protein 1	Vaccine	I	12	Maximum-tolerated dose; Incidence of adverse events	NCT03865212
MGD013	PD-1; LAG-3	I	92	ORR	NCT04653038
Ceralasertib alone vs Ceralasertib Durvalumab	PD-(L)1; ataxia telangiectasia and Rad3-related kinase	II	195	Changes in CD8 + T cell infiltration of tumours induced by ceralasertib monotherapy; ORR	NCT05061134
Pembrolizumab plus Lenvatinib	PD-1; VEGFR1, VEGFR2, VEGFR3, FGFR1-4, PDGFRα, KIT, RET	III	660	PFS; OS	NCT03820986
YH003 Plus Pebolizumab and Albumin Paclitaxel	CD-40; PD-1; Microtubules	II	43	ORR	NCT05420324
Vactosertib plus Pembrolizumab	PD-1; activin receptor-like kinase 5	II	30	Overall Response Rate	NCT05436990
Olaparib	BRCA1/2 Genes	II	15	ORR	NCT05482074
Intrathecal nivolumab, plus or not intravenous nivolumab in treating patients with leptomeningeal disease	PD-1;	I	50	Safety and/or recommended dose of intrathecal of Nivolumab	NCT03025256
Binimetinib and Encorafenib plus Nivolumab vs Ipilimumab plus Nivolumab	BRAF-V600; PD-1; CTLA-4	II	112	PFS	NCT04511013
Apatinib plus Temozolomide	VEGFR2; DNA	II	30	PFS	NCT03422445
Dinaciclib	CDK	II	72	1-year overall survival rate	NCT00937937
Vorolanib plus toripalimab	VEGFR/PDGFR; PD-1	II	40	ORR	NCT03602547

*CTLA-4* cytotoxic T-Lymphocyte Antigen 4; *DLT* dose-limiting toxicities; *DFS* disease-free survival; *FGFR1-4* fibroblast growth factor receptor 4; *HIF1* hypoxia-inducible factor 1-alpha; *IMRT* intensity modulation radiation therapy; *IL-2R* interleukin-2 Receptor; *KIT* stem cell factor receptor; *LAG3* lymphocyte-activation gene 3; *MDM2* murine double minute 2; *NA* not applicable; *ORR* objective response rate; *SO* overall survival; *PDGFRα* platelet-derived growth factor alfa; *PDGFRβ* platelet-derived growth factor receptor beta; *PD-(L)1* programmed death-(ligand)1; *PFS* progression-free survival; *RET* rearranged during transfection; *RFS* recurrence-free survival; *VEGF* vascular-endothelial growth factor

Furthermore, the angiogenesis signatures might be considered an applicable biomarker for anti-VEGF plus immunotherapy, although VEGF signaling has an important immune-modulating function in the microenvironment [80]. Likewise, pembrolizumab plus Lenvatinib (another vascular endothelial growth factor receptor inhibitor) revealed a potential anticancer effect in patients with advanced melanoma. In the LEAP-003 trial, this combination is currently ongoing also for patients with acral and mucosal melanomas [81]. Similar minor clinical studies of combination treatments for mucosal melanoma are also underway and recruiting patients, as well as trials with Immune checkpoint inhibitors (ICI) or apatinib (anti-VEGFR2) plus temozolomide (alkylating agent similar to dacarbazine) [NCT03422445]; radiation plus ICI in mucosal melanoma of the head and neck [NCT04318717]; Camrelizumab (an anti-PD1), plus anlotinib (multitargeted tyrosine kinase inhibitor), and nab-paclitaxel. Promising combinations with ICI under evaluation include MDM2 inhibitor (APG-115) and aldesleukin (NCT03611868, NCT02748564) or single-agent nemvaleukin (NCT04830124). Nevertheless, most of these studies enrolled Asian patients, most frequently affected by this neoplasm. In MM, CDK4 amplification and/or CCND1 amplification and/or p16 (CDKN2A) loss leads to the dysregulation of cell cycle progression, which justifies the rationale of targeting CDK4/6 in some preclinical and clinical trials [82]. Another intriguing target for MM is ataxia telangiectasia and rad3-related (ATR) kinase. An orally available morpholino-pyrimidine-based inhibitor of this molecule is investigated in a phase 2 trial in combination with anti-PD-L-1 in unresectable or advanced melanoma and primary or secondary resistance to PD-(L)1 inhibition, to date an unmet clinical need [NCT05061134].

Hence, even if the combination treatments could enhance long-term outcomes in this rare and aggressive subtype of melanoma, we must consider clinic-pathological features and mutational profiles of a single patient to open the road for novel treatment targets [83]. In this scenario, where traditional therapies are less effective, further prospective umbrella clinical trials are critical to promoting precision medicine and inclusion of the non-Asian population, despite the higher incidence in Orient.

To date, several clinical studies conducted for neoadjuvant and adjuvant therapies are focused on exploring an innovative strategy, dealing with this tumor in the earliest setting.

## Data Availability

Data will be made available on request.

## Abbreviations

MM: Mucosal melanoma
CM: Cutaneous melanoma
KIT: KIT Proto-Oncogene, Receptor Tyrosine Kinase
BRAF: V-RAF murine sarcoma viral oncogene homolog B1
MAPK: Mitogen-activated protein kinase
NRAS: Neuroblastoma ras viral oncogene homolog
NO: Nitric oxide
MSH: Melanocyte Stimulating Hormone
PD-L1: Programmed death-ligand 1
PD-1: Programmed death 1
GNAQ: Guanine nucleotide-binding protein G(q) subunit alpha
GNA11: Guanine nucleotide-binding protein alpha-11
MEK: Mitogen-activated extracellular signal-regulated kinase
VEGF: Vascular endothelial growth factor
CTLA4: The cytotoxic T lymphocyte 4
SLNB: Sentinel lymph node biopsy
ORR: Objective response rate
PFS: Progression Free Survival
OS: Overall Survival
NF1: Neurofibromin 1
ICI: Immune checkpoint inhibitors
MDM2: Murine double minute‑2
CDKN2A: Cyclin-dependent kinase inhibitor 2A
CDK: Cyclin-dependent kinases
CCND1: Cyclin D1
TMB: Tumor mutational burden
ATR: Ataxia telangiectasia and rad3-related
